# Chronic Respiratory Disease Prevalence, Burden, and Treatment in Cape Town: A Cross‐Sectional Study

**DOI:** 10.1002/hsr2.70992

**Published:** 2025-07-09

**Authors:** Marie Stolbrink, Lisanne Liebenberg, Tantaswa Ndlelana, Kischa Petersen, Stephanie Griffith‐Richards, Kevin Mortimer, Brian Allwood

**Affiliations:** ^1^ Department of Lung Health and Tuberculosis, Clinical Sciences Liverpool School of Tropical Medicine Liverpool UK; ^2^ Division of Pulmonology, Department of Medicine Stellenbosch University Stellenbosch South Africa; ^3^ Radiology Department Tygerberg Academic Hospital Cape Town South Africa; ^4^ Cambridge Africa, Department of Pathology University of Cambridge Cambridge UK; ^5^ School of Clinical Medicine, College of Health Sciences University of KwaZulu‐Natal Durban South Africa; ^6^ Liverpool University Hospitals NHS Foundation Trust Liverpool UK; ^7^ Department of Pulmonology Tygerberg Hospital Cape Town South Africa

**Keywords:** anti‐asthmatic agents, asthma, chronic obstructive pulmonary disease, eosinophilia, exacerbation, hospitalisation, low‐ and middle‐income countries

## Abstract

**Background and Aims:**

There is a high burden of respiratory symptoms in South Africa, but accurate classification of diagnoses is challenging. Due to the adverse risk profile, inhaled corticosteroid (ICS) treatment should only be used in specific conditions, such as asthma or chronic obstructive pulmonary disease (COPD) with eosinophilia. Those with tuberculosis or who are HIV‐positive may be at particular risk. Hence, establishing underlying diagnoses and indications for ICS treatment is crucial. The primary aim was to establish the underlying diagnosis after investigations, the secondary aim was to identify any indication for ICS treatment (asthma or eosinophilia).

**Methods:**

We conducted a cross‐sectional study of adults attending two hospitals in Cape Town, South Africa, with exacerbations of chronic respiratory diseases. Participants performed investigations at least 8 weeks after recruitment. Questionnaires and extensive investigations (full lung function with bronchodilator reversibility, allergy tests, computed tomography) were completed.

**Results:**

Between 2021 and 2023, a total of 224 participants were recruited, of whom 101 attended investigations, yet 40 died before they could attend. Most were current (49) or ex‐smokers (40). A total of 46 previously had pulmonary tuberculosis. Post‐tuberculosis lung disease (PTLD) with COPD (28) or COPD alone (27) were the most common diagnoses. Ten participants had asthma. Serum eosinophilia was present in 19 participants. There was no clear indication for ICS treatment for 5/7 participants who used ICS, and 22/30 who used ICS‐long‐acting beta‐agonists combination.

**Conclusion:**

COPD and PTLD were more common, asthma and eosinophilia were less prevalent than expected. There was a high symptom burden. Modifiable risks (tuberculosis, smoking) were also common. ICS treatment appeared to be independent of underlying diagnosis and could lead to harm. Approximately 1 in 5 died shortly after admission. Strategies to better serve this patient group are urgently needed.

## 1 Introduction

Low‐ and middle‐income countries (LMICs) have a high burden of non‐communicable, chronic respiratory diseases (CRDs) [1]. Airways diseases, such as asthma, chronic obstructive pulmonary disease (COPD), bronchiectasis, or post‐tuberculosis lung disease (PTLD), form a large proportion of CRDs in this setting. South Africa has one of the highest asthma mortality rates worldwide, and up to a quarter of the population has COPD [2, 3, 4].

Whilst different airways diseases have similar symptoms, it is important to establish the underlying diagnosis, as the pathophysiology, prognosis, and treatments differ [5]. Inhaled corticosteroids (ICS) are indicated for COPD patients with blood eosinophilia and all asthma patients [6, 7]. However, there are risks associated with ICS, e.g., pneumonia, systemic side effects, HIV drug interactions, and tuberculosis infection [8, 9, 10]. South Africa has one of the highest prevalences of HIV and tuberculosis globally [11]. Establishing CRD diagnoses and optimizing treatments in this population is therefore especially important. Accurate diagnoses may also have impacts on understanding the burden of diseases, and hence ultimately resource allocation.

CRD exacerbations are a driver of disease burden, those who require treatment in hospitals are at increased risk of morbidity and mortality, and exacerbations also cause significant healthcare costs [6, 7].

This study aimed to (1) describe the underlying diagnoses of patients with airways disease in Cape Town hospitals, and (2) establish the proportion of patients with airways disease with asthma or eosinophilia who might potentially benefit from ICS.

## 2 Methods

### 2.1 Setting

We conducted a cross‐sectional study of adults in two hospitals in Cape Town, South Africa: Tygerberg Hospital (TBH), a large tertiary public hospital, and Khayelitsha District Hospital (KDH), a public hospital located in an informal settlement. Participants were recruited when they attended the hospital between November 2021 and March 2023 and were investigated at a study visit ≥ 8 weeks later. Ethical approval was gained from Stellenbosch University (N20/11/124), Liverpool School of Tropical Medicine (20‐094), and Western Cape Department of Health. All participants gave written consent before participating in the study. We followed EQUATOR Network guidelines (Supporting Information S1: Data S1).

### 2.2 Participants

All new medical patients were screened within 72 h of admission and recruited randomly if eligible. Adults (≥ 18 years) were recruited if they attended primarily with an airways disease exacerbation, defined as requiring treatment with bronchodilators and corticosteroids. The diagnosis of airways disease was taken from the medical notes. Those who did not present with an exacerbation, on treatment for or suspected of active tuberculosis or malignancy (as per the treating medical team), with contraindications for spirometry or computed tomography (e.g., pregnancy), and those with conditions likely to impair interpretation of results were excluded. All potential participants were discussed with an experienced respiratory physician (MS) before recruitment.

### 2.3 Variables

Demographic and clinical details were collected using a standardized form at recruitment. At the study visit, participants completed questionnaires (St. George's Respiratory Questionnaire (SGRQ), EuroQol‐5D (EQ‐5D), serum and sputum eosinophil analysis, total and specific immunoglobulin E (IgE), exhaled nitric oxide (FeNO) measurement, full lung function studies with bronchodilator‐reversibility and diffusion capacity, and 6‐min walk test [12, 13, 14]. Inspiratory and expiratory high‐resolution computed tomography (HRCT) was completed at NuMeRi, Stellenbosch University, if indicated by symptoms of bronchiectasis (at least two of chronic cough, chronic sputum production, or at least two respiratory infections per year) and if no CT had been completed in the previous year [15].

HRCTs were reported according to a standardized data collection form by a local expert radiologist (SGJ). Lung function tests were conducted in a fully equipped lung function laboratory at Tygerberg Hospital by trained medical technologists. All results were compared to normal values using the Global Lung Function Initiative [16]. Bronchodilator reversibility was defined by improvement in forced expiratory volume in one second (FEV1) of ≥ 12% and > 200 mL [6]. Serum eosinophilia was defined as serum eosinophil count > 300 cells/microlitre. Initial diagnoses, medications, readmission, and death recordings were taken from the medical notes. Post‐investigations diagnoses were made according to specified criteria (Table 1). Participants could have multiple diagnoses.

**Table 1:** Definitions of post‐investigation diagnoses.

Diagnosis	Criteria
Asthma	Symptom variability ± bronchodilator‐reversibility in FEV1 (≥ 12% and > 200 mL) [6]
COPD	FEV1/forced vital capacity (FEV1/FVC) < 0.7 and significant smoking history (≥ 20 pack years or smoking substances other than tobacco) or emphysema on CT [7]
PTLD	History of tuberculosis ± CT findings in keeping with previous tuberculosis (fibrocavitary change, upper lobe fibrosis, cavitation)
Bronchiectasis	Chronic productive cough ± CT findings of bronchiectasis [15]

Abbreviations: COPD, chronic obstructive pulmonary disease; CT, computed tomograph; FEV1, forced expiratory volume in one second (FEV1); PTLD, post‐tuberculosis lung disease.

The prevalence of asthma or eosinophilia in this population was unknown. Recruitment of 220 participants would allow a 95% confidence level for an estimated prevalence of 60% with 10% precision, with a 10% loss to follow‐up. Data were collected using RedCap hosted at Stellenbosch University [17]. Data were analyzed based on the type and distribution of data using R, version 4 [18]. Mann–Whitney U or Spearman's correlation was used for correlation analyses, Mann–Whitney U, Fisher's exact test, or Chi‐square for univariate analyses. Univariate analyses were conducted for clinically plausible variables affecting serum eosinophilia and bronchodilator reversibility. All tests were two‐sided. The statistically significant *p*‐value was < 0.05.

The primary outcome was to describe the underlying diagnoses after study investigations. The secondary outcome was diagnosis of asthma or serum eosinophilia.

## 3 Results

In total, 224 participants (155 from TBH, 69 from KDH) were recruited. Of these 40 (18%) died before they could perform investigations, 42 were lost to follow‐up, 19 withdrew consent, 18 developed exclusion criteria, and 4 were duplicate entries (Supporting Information S1: Data S1).

### 3.1 Demographics

A total of 101 participants (67 from TBH, 35 from KDH) completed investigations. A total of 51 (51%) were female with a median age of 59 years (Table 2). A total of 61 (60%) had hypertension, and 21 (21%) were HIV‐positive. A total of 46 (46%) previously had pulmonary tuberculosis, and 24 had it more than once. Most (70, 69%) had no allergic co‐morbidities, but 46 (46%) reported a family history of asthma or allergy. Most had a smoking history, 49 were current, and 40 were ex‐smokers, with a median of 17.8 pack years (IQR: 8.7–31.1). A total of 90 (89%) experienced exacerbations in the previous year, with a median of 3 exacerbations. A total of 44/101 participants were re‐admitted for a CRD exacerbation in the study period.

**Table 2:** Demographics of recruited participants and those who attended investigations.

	All recruited *N* (%)	Those who attended investigations *N* (%)
	*n* = 220	*n* = 101
Location		
TBH	155 (70.5)	67 (66.3)
KDH	69 (29.5)	34 (33.7)
Sex		
Female	107 (48.6)	51 (50.5)
Male	113 (51.4)	50 (49.5)
Age (years)		
Median (IQR)	59 (49–65)	59 (49–64)
Range	18–87	22–87
Leaving school age (years)		
Median (IQR)	16 (15–18)	17 (15–18)
Range	8–30	8–30
Employment		
Employed	31 (14.1)	15 (14.8)
Pensioner	96 (43.6)	43 (42.6)
Unemployed	93 (42.3)	43 (42.6)
Smoking		
Never	31 (14.2)	13 (12.9)
Ex	87 (39.9)	40 (39.6)
Current	100 (45.9)	48 (47.5)
	Missing: 2	Missing: 0
Pack years smoked		
Median	20.1 (8.7–37)	17.8 (8.7–31.1)
Range	0.8–108	1–92
	Missing: 1	Missing: 0
Admission diagnosis (multiple answers possible)		
Asthma	81	41
PTLD	20	11
COPD	129	56
Bronchiectasis	11	4
Unknown	5	3
Inhalers used (multiple answers possible)	*n* = 193	*n* = 94
SABA	171 (88.6)	80 (86.0)
ICS	20 (10.4)	9 (9.6)
ICS‐LABA	59 (30.6)	30 (31.9)
Had exacerbations in last year (self‐reported)	190 (86.4)	90 (89.1)
Number of exacerbations in last year (self‐reported)		
Median (IQR)	2 (2–6)	3 (2–6)
Attended hospital for CRD exacerbation in last		
12 Months	161 (80.5)	76 (83.5)
HIV status		
Negative	175 (79.6)	80 (79.2)
Positive	40 (18.2)	21 (20.8)
Unknown	2 (2.3)	
On anti‐retroviral therapy (% of those positive)	33/40 (82.5)	19/21 (90.4)
History of tuberculosis (self‐reported)		
Yes	98 (44.6)	46 (45.5)
No	119 (54.1)	54 (53.5)
Unknown	3 (1.4)	1 (1.0)
Number of times treated for tuberculosis (self‐reported)		
1	43 (43.9)	20 (43.5)
2	25 (25.5)	11 (23.9)
≥ 3	28 (28.5)	13 (28.2)
Unknown	2 (2.0)	2 (4.4)
Co‐morbidities (multiple answers possible)		
Diabetes	42 (19.1)	17 (16.8)
Hypertension	132 (60.0)	61 (60.4)
Cardiac disease	48 (21.8)	17 (16.8)
Obesity	17 (7.7)	8 (7.9)
Smoking substances other than tobacco	37 (16.8)	14 (13.9)
Allergic co‐morbidities (multiple answers possible)		
Allergy	20 (9.1)	12 (11.9)
Eczema	21 (9.5)	10 (9.9)
Hayfever	21 (9.5)	12 (11.9)
Anaphylaxis	1 (0.5)	1 (1.0)
Rhinitis	22 (10.0)	14 (13.9)
Family history of asthma or allergy	88 (40.0)	46 (45.5)
Hospital readmissions within study period	89 (40.5)	44 (43.6)

*Note:* Number (%) unless otherwise indicated.

Abbreviations: 95% CI, 95% confidence interval; HIV, human immunodeficiency virus; ICS, inhaled corticosteroid; ICS‐LABA, ICS‐long‐acting beta‐agonist combination; IQR, inter‐quartile range; KDH, Khayelitsha District Hospital; PTLD, post‐tuberculosis lung disease; SABA, short‐acting beta‐agonist inhaler; TBH, Tygerberg Hospital.

### 3.2 Symptom Burden

All participants who attended for investigations completed the questionnaires. Most had an mMRC dyspnoea score of 4 (64, 63%, Table 3). The mean SGRQ score was 64.7 (95% CI: 60.2–69.2), where 100 is worst health. Only 10 (10%) participants reported perfect health on the EQ‐5D, almost a quarter (24, 23.8%) reported severe problems with mobility, and at least some impairment when conducting usual activities.

**Table 3:** Results from burden of disease questionnaires.

Attribute	*N* (%)
mMRC Breathlessness score	
0	9 (8.9)
1	3 (3.0)
2	8 (7.9)
3	17 (16.8)
4	64 (63.4)
St George's Respiratory Questionnaire (SGRQ)	
Symptoms score	
Mean (95% CI)	63.60 (59.47–67.73)
Activity score	
Mean (95% CI)	76.86 (71.58–82.14)
Impacts score	
Mean (95% CI)	58.07 (53.19–62.94)
Total score	
Mean (95% CI)	64.68 (60.21–69.15)
EuroQol‐5D questionnaire (EQ‐5D)	
Visual analogue health score	
Mean (95% CI)	60.6 (56.4–64.7)
EQ5D Value Index (0 = “worst health”, 1 = “perfect health”)	
Mean (95% CI)	0.59 (95% CI: 0.54–0.65)
Number with “perfect health”	10 (9.9)

*Note: N* = 101.

Abbreviations: 95% CI, 95% confidence interval; EQ‐5D, EuroQol‐5D questionnaire; IQR, interquartile range; mMRC, modified Medical Research Council dyspnoea scale.

### 3.3 Eosinophilic Inflammation and Atopy

**Figure 1:**
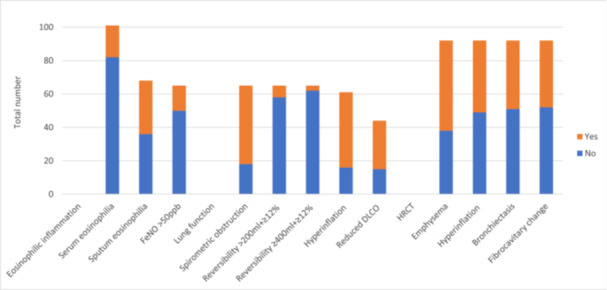
Prevalence of markers of eosinophilic inflammation, lung function, and radiological abnormalities. Total number: participants who completed the investigation. Serum eosinophilia: serum eosinophils ≥ 300 cells/microlitre; FeNO: exhaled nitric oxide; spirometric obstruction: FEV1/FVC < lower limit of normal (LLN); Reversibility: change in FEV1 after bronchodilator administration; hyperinflation: RV/TLC > upper limit of normal; DLCO: Transfer factor corrected for hemoglobin; Reduced DLCO: DLCO < LLN; HRCT, high‐resolution computed tomography.

A total of 19 (19%) participants had a peripheral blood eosinophil count of ≥ 300 cells/microlitre (Figure 1). Of the 68 participants who provided sputum, 32 (46%) had sputum eosinophils. FeNO was successfully measured in 65 (64%) participants, with a median reading of 20 ppb (IQR: 13–41 ppb, Supporting Information S1: Data S1). A total of 15 (23%) had a FeNO level of > 50 ppb.

A total of 50 (50%) had a total IgE of > 100 IU/mL, and 47 (47%) tested positive on common aeroallergen panel. The most common aeroallergen reactivity was for house dust mite (Supporting Information S1: Data S1).

### 3.4 Physiological Test Results

The mean 6‐min walking distance (6MWD) by the 72 participants who completed the test was 368 m (95% CI: 341–395 m, Supporting Information S1: Data S1).

All participants performed spirometry. Bronchodilator‐reversibility testing was conducted for 65 participants, 19 only had pre‐bronchodilator and 17 had only post‐bronchodilator spirometry testing (Supporting Information S1: Data S1). For those participants with bronchodilator‐reversibility testing, the mean post‐bronchodilator FEV1 was 56% predicted (95% CI: 32%–73%) and the mean FVC was 79% predicted (95% CI: 74%–84%, Supporting Information S1: Data S1). Obstruction according to the lower limit of normal (LLN) was present in 47 (72%) participants (Figure 1). Seven (11%) participants had an improvement of > 200 mL and ≥ 12% in FEV1 after bronchodilator. Three of these had an improvement of ≥ 12% and ≥ 400 mL in FEV1, highly suggestive of asthma.

### 3.5 Body Plethysmography

Full lung function tests were conducted in 83 participants. Pre‐ and post‐bronchodilator tests were conducted in 61 participants, 7 only had pre‐ and 15 only post‐bronchodilator tests (Supporting Information S1: Data S1). For those with bronchodilator‐reversibility results, hyperinflation, or air‐trapping (raised residual volume/total lung capacity [RV/TLC]) was present in 45/61 (74%) by being greater than the upper limit of normal (ULN, Figure 1). Restriction (TLC < LLN) was present in 7/61 (12%) participants.

The transfer factor (DLCO), corrected for hemoglobin, was measured in 44 participants. 29 participants (66%) had a DLCO less than LLN (Supporting Information S1: Data S1).

### 3.6 Radiological Findings

HRCTs were conducted in 70 participants as part of the study, and an additional 22 had CTs in the previous year. The most common finding on CT was emphysema in 54 participants (59%), followed by hyperinflation in 43 (47%) and bronchiectasis in 41 (45%, Figure 1, Supporting Information S1: Data S1).

### 3.7 Diagnoses

**Figure 2:**
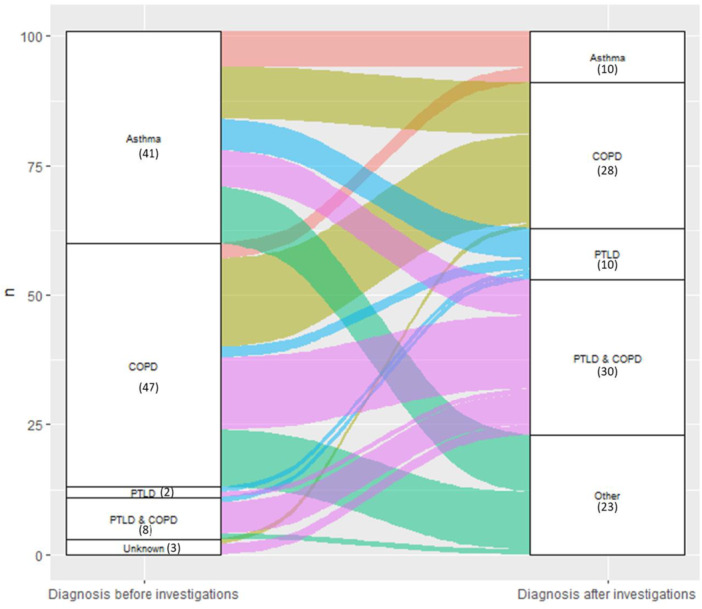
Sankey chart of grouped diagnoses before and after investigations. *N* = 101; diagnoses were grouped by prevailing diagnosis; number with diagnosis in brackets. COPD, chronic obstructive pulmonary disease; PTLD, post‐tuberculosis lung disease.

The commonest admission diagnoses were COPD and asthma (56 and 41 participants, respectively, Table 1). The diagnosis of 71 (70%) participants changed after investigations (Supporting Information S1: Data S1). Most moved from a diagnosis of COPD to a diagnosis of COPD with PTLD (12 participants) or from a diagnosis of asthma to a COPD diagnosis (9 participants, Figure 2). Ten participants had a final diagnosis of asthma. Almost all of those who had a history of tuberculosis infection received a diagnosis of PTLD after investigations (45/46 participants).

### 3.8 Treatment

**Figure 3:**
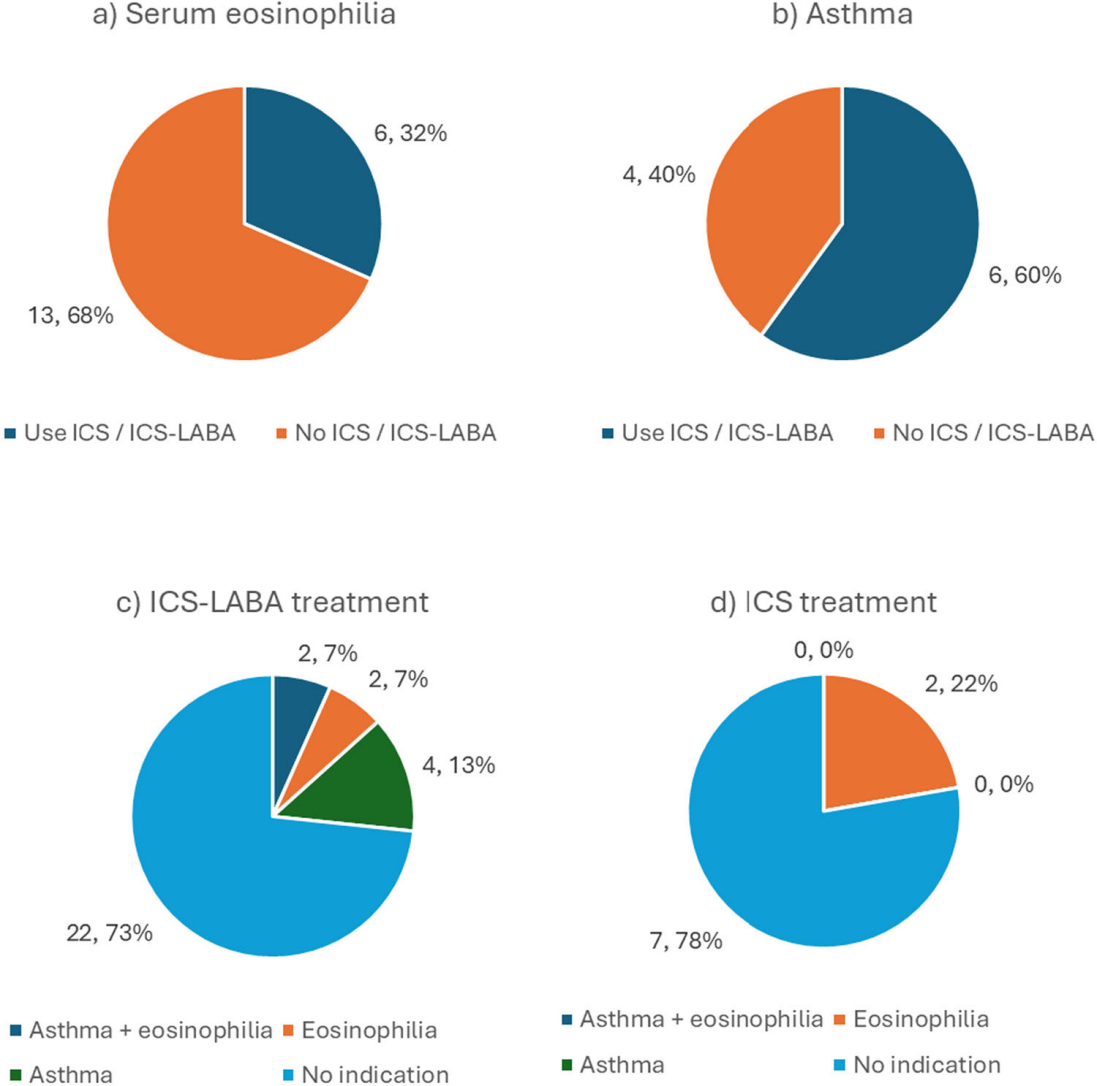
ICS‐containing treatments in participants with (a) serum eosinophilia, (b) asthma diagnosis; and indications for ICS‐containing treatments in those receiving (c) ICS‐LABA or (d) ICS. Total number, percentage, with variable. Eosinophilia: serum eosinophils ≥ 300 cells/microlitre.

A total of 94 participants regularly used inhaled medicines. Most (58, 57%) only used one inhaler (Table 1). SABA was used by 80 (85%), ICS by 9 (10%), and ICS‐LABA by 30 (32%). Of those who had serum eosinophilia, 2/19 used ICS and 4/19 used ICS‐LABA (Figure 3). Of the 10 who had a post‐investigation asthma diagnosis, six used ICS‐LABA, and none used ICS alone.

### 3.9 Further Statistical Analysis

In univariate analysis, a raised serum eosinophil count was significantly associated with male sex, total IgE, and a positive aeroallergen screen (*p* = 0.02, *p* = 0.008, and *p* = 0.008, respectively, Supporting Information S1: Data S1). No clinically plausible variables that may affect bronchodilator‐reversibility (FEV1 improvement > 200 mL and ≥ 12%) were statistically significant in univariate analysis (Supporting Information S1: Data S1).

## 4 Discussion

COPD and PTLD were the most common diagnoses in adult patients attending hospitals with airways disease exacerbations in Cape Town. Approximately 1 in 5 died, and 40% were readmitted during the study period.

There was a high burden of symptoms and reduced quality of life with raised healthcare utilization, 89% of participants had an exacerbation in the previous year, and 84% attended a hospital for them at least once. Smoking, tuberculosis, spirometric obstruction, and hyperinflation were common. Only 10 participants received a final diagnosis of asthma, and 19 participants had blood eosinophilia. This is in stark contrast to the reported around 20% community prevalence of asthma in South African teenagers and the reported high asthma mortality [2, 4].

Only those with asthma or COPD with raised serum eosinophils should receive ICS treatment [6, 7, 19]. Yet of those who had serum eosinophilia, only about a third used ICS‐containing inhalers, and only 6/10 of those with asthma used any ICS. Use of ICS has been associated with multiple risks, including mycobacterial disease, yet for 5/7 participants who used ICS and 22/30 who used ICS‐LABA, there was no clear indication for ICS treatment [20]. Given the risks of ICS use, large‐scale, randomized studies are needed to explore the safety of ICS in this high‐burden setting [8, 9, 10]. Studying the efficacy of alternative treatment protocols and comprehensive studies examining the systemic effects of tuberculosis on respiratory health would also be beneficial.

An important but unexpected finding was the very high mortality (40/224, 18%), which is likely to be an underestimate as the majority of these were in‐hospital deaths and without access to community death records. There are limited data for mortality after hospital admissions for CRDs in Africa. In Europe, 5% of those admitted with COPD died, and a PTLD study in Malawi demonstrated 3% mortality 1 year after tuberculosis treatment completion [21, 22]. Exploratory data analysis revealed that a COPD or PTLD diagnosis, cardiac disease, higher smoking burden, and no SABA use were significantly associated with death (Supporting Information S1: Data S1). There was also an incidental finding of high re‐admission rates: 44% of participants were re‐admitted for another exacerbation within a median of 90 days. Exploratory analysis indicated that employment status, exacerbation history, SABA use, and previous tuberculosis were associated (Supporting Information S1: Data S1). Further studies in this area are needed to reduce morbidity and mortality.

We acknowledge the limitations of this study. There was a high loss to follow up. However, this is in keeping with other findings from the Western Cape province, for example, in HIV clinics [23]. Yet the populations of those who completed investigations and those who were recruited were very similar. Other causes of eosinophilia exist, but this is not considered in international or local guidance, and ICS treatment for those with respiratory symptoms is advised regardless. There was reliance on self‐reported data for some variables, such as the previous exacerbation number. The participants were recruited from secondary or tertiary care hospitals, and hence, there may be selection bias towards more serious and complex diseases. This may impact reproducibility in other settings, such as primary care. We may have missed potential participants who only spent a short time in the hospital. We were limited by the number who were able to complete bronchodilator spirometry. Participants were assessed at a single timepoint with the design limitations inherent in a cross‐sectional study, including the challenges to determine causality.

The strengths of the study include that participants received extensive investigations and assessments, which would otherwise be unavailable in this setting, including body plethysmography, FeNO, and CT scans, to establish a diagnosis. This is the first time that such investigations have been conducted on this population. We used established criteria for defining post‐investigation diagnoses.

Further work is needed to establish the risks and benefits of ICS use in those without evidence of asthma or raised eosinophils in settings with high tuberculosis incidence, especially in those with PTLD. More studies are needed on the prognosis of those in the hospital with airways diseases. Many LMICs, and especially more rapidly growing economies, share South Africa's high burden of tuberculosis, CRDs, and a growing smoking prevalence, so it would be useful to establish whether our findings can be replicated, including primary care [24].

Despite many participants being labeled as “asthma” initially, PTLD and COPD were more common after investigations, with possible impacts on treatments and public health resource allocation. ICS use seemed to be unrelated to blood eosinophilia or asthma diagnosis. Education of patients and practitioners, and control of tuberculosis and smoking are particularly important to control and prevent airways disease. There was high mortality and high disease burden, and most had at least one modifiable risk factor for airways disease. Strategies to better serve this patient group are urgently needed.

## Author Contributions

M.S., K.M., and B.A. designed the study. M.S., L.L., T.N., and K.P. performed the study and collected data. M.S., K.M., and B.A. analyzed data and wrote the manuscript. S.G.R. analyzed the CT scans. All authors commented on the final version of the manuscript. All authors have read and approved the final version of the manuscript. M.S. had full access to all of the data in this study and takes complete responsibility for the integrity of the data and the accuracy of the data analysis.

## Ethics Statement

Ethical approval was gained from Stellenbosch University (N20/11/124), Liverpool School of Tropical Medicine (20‐094), and Western Cape Department of Health.

## Consent

All participants gave written consent before participating in the study.

## Conflicts of Interest

The authors declare no conflicts of interest.

## Transparency Statement

The lead author, Marie Stolbrink, affirms that this manuscript is an honest, accurate, and transparent account of the study being reported; that no important aspects of the study have been omitted; and that any discrepancies from the study as planned (and, if relevant, registered) have been explained.

## Supporting information

Health Sci Rep Supporting data 10.

## Data Availability

The data that support the findings of this study are available from the corresponding author upon reasonable request.
