# Supplementary material for: Chronic Respiratory Disease Prevalence, Burden, and Treatment in Cape Town: A Cross‐Sectional Study
Source: Health Sci Rep. 2025 Jul 9;8(7):e70992. doi: 10.1002/hsr2.70992 (PMC12239511; doi:10.1002/hsr2.70992)
Supplement: Supplementary file 1 — Health Sci Rep Supporting data 10. [file HSR2-8-e70992-s001.docx]

Supplementary data

Table of Contents

[Supplementary Table 1: STROBE (EQUATOR) Criteria 1](#_Toc190092732)

[Supplementary Table 2: EQ-5D detailed results 3](#_Toc190092733)

[Supplementary Table 3: Number completing physiological tests 3](#_Toc190092734)

[Supplementary Table 4: DLCO results 4](#_Toc190092735)

[Supplementary Table 5: FeNO results 4](#_Toc190092736)

[Supplementary Table 6: Laboratory results 5](#_Toc190092737)

[Supplementary Table 7: Correlations 6](#_Toc190092738)

[Supplementary Table 8: Univariate analysis for serum eosinophil count >300 cells/microlitre 6](#_Toc190092739)

[Supplementary Table 9: Univariate analysis for FEV1 improvement after bronchodilator administration 8](#_Toc190092740)

[Supplementary Table 10: Univariate analysis for PEF improvement after bronchodilator administration 10](#_Toc190092741)

[Supplementary Table 11: Univariate analysis for death 12](#_Toc190092742)

[Supplementary Table 12: Univariate analysis for re-admissions 13](#_Toc190092743)

[Supplementary Figure 1: Number of diagnoses pre- and post-investigations 15](#_Toc190092744)

# Supplementary Table 1: STROBE (EQUATOR) Criteria

Information on the STROBE Initiative is available at [www.strobe-statement.org](http://www.strobe-statement.org).

STROBE Statement—Checklist of items that should be included in reports of ***cross-sectional studies***

|  | Item No | Recommendation | Page |
| --- | --- | --- | --- |
| **Title and abstract** | 1 | (*a*) Indicate the study’s design with a commonly used term in the title or the abstract | 1,3 |
|  |  | (*b*) Provide in the abstract an informative and balanced summary of what was done and what was found | 3 |
| Introduction | | |  |
| Background/rationale | 2 | Explain the scientific background and rationale for the investigation being reported | 4 |
| Objectives | 3 | State specific objectives, including any prespecified hypotheses | 4 |
| Methods | | |  |
| Study design | 4 | Present key elements of study design early in the paper | 4-5 |
| Setting | 5 | Describe the setting, locations, and relevant dates, including periods of recruitment, exposure, follow-up, and data collection | 4-5 |
| Participants | 6 | (*a*) Give the eligibility criteria, and the sources and methods of selection of participants | 4-5 |
| Variables | 7 | Clearly define all outcomes, exposures, predictors, potential confounders, and effect modifiers. Give diagnostic criteria, if applicable | 4-5 |
| Data sources/ measurement | 8* | For each variable of interest, give sources of data and details of methods of assessment (measurement). Describe comparability of assessment methods if there is more than one group | 4-5 |
| Bias | 9 | Describe any efforts to address potential sources of bias | 4-5 |
| Study size | 10 | Explain how the study size was arrived at | 4-5 |
| Quantitative variables | 11 | Explain how quantitative variables were handled in the analyses. If applicable, describe which groupings were chosen and why | 4-5 |
| Statistical methods | 12 | (*a*) Describe all statistical methods, including those used to control for confounding | 4-5 |
|  |  | (*b*) Describe any methods used to examine subgroups and interactions | 4-5 |
|  |  | (*c*) Explain how missing data were addressed | 4-5 |
|  |  | (*d*) If applicable, describe analytical methods taking account of sampling strategy | N/A |
|  |  | (*e*) Describe any sensitivity analyses | N/A |
| Results | | |  |
| Participants | 13* | (a) Report numbers of individuals at each stage of study—eg numbers potentially eligible, examined for eligibility, confirmed eligible, included in the study, completing follow-up, and analysed | 5-7 |
|  |  | (b) Give reasons for non-participation at each stage | 5-7 |
|  |  | (c) Consider use of a flow diagram | Supp data |
| Descriptive data | 14* | (a) Give characteristics of study participants (eg demographic, clinical, social) and information on exposures and potential confounders | 5-7 |
|  |  | (b) Indicate number of participants with missing data for each variable of interest | 5-7 |
| Outcome data | 15* | Report numbers of outcome events or summary measures | 8-13 |
| Main results | 16 | (*a*) Give unadjusted estimates and, if applicable, confounder-adjusted estimates and their precision (eg, 95% confidence interval). Make clear which confounders were adjusted for and why they were included | 8-13 |
|  |  | (*b*) Report category boundaries when continuous variables were categorized | 8-13 |
|  |  | (*c*) If relevant, consider translating estimates of relative risk into absolute risk for a meaningful time period | N/A |
| Other analyses | 17 | Report other analyses done—eg analyses of subgroups and interactions, and sensitivity analyses | 8-13 |
| Discussion | | |  |
| Key results | 18 | Summarise key results with reference to study objectives | 14-15 |
| Limitations | 19 | Discuss limitations of the study, taking into account sources of potential bias or imprecision. Discuss both direction and magnitude of any potential bias | 14-15 |
| Interpretation | 20 | Give a cautious overall interpretation of results considering objectives, limitations, multiplicity of analyses, results from similar studies, and other relevant evidence | 14-15 |
| Generalisability | 21 | Discuss the generalisability (external validity) of the study results | 14-15 |
| Other information | | |  |
| Funding | 22 | Give the source of funding and the role of the funders for the present study and, if applicable, for the original study on which the present article is based | 2,15 |

*Give information separately for exposed and unexposed groups.

# Supplementary Table 2: EQ-5D detailed results

| **EQ-5D** | N (%) |
| --- | --- |
| **Mobility**  No problem  Slight problem  Moderate problem  Severe problem  Unable to / extreme problem | 29 (28.7)  23 (22.8)  19 (18.8)  24 (23.8)  6 (5.9) |
| **Self-care**  No problem  Slight problem  Moderate problem  Severe problem  Unable to / extreme problem | 51 (50.5)  12 (11.9)  15 (14.9)  15 (14.9)  8 (7.9) |
| **Usual Activities**  No problem  Slight problem  Moderate problem  Severe problem  Unable to / extreme problem | 29 (28.7)  17 (16.8)  17 (16.8)  19 (18.8)  19 (18.8) |
| **Pain / Discomfort**  No problem  Slight problem  Moderate problem  Severe problem  Unable to / extreme problem | 44 (43.6)  25 (24.8)  12 (11.9)  17 (16.8)  3 (3.0) |
| **Anxiety / Depression**  No problem  Slight problem  Moderate problem  Severe problem  Unable to / extreme problem | 46 (45.5)  29 (28.7)  4 (4.0)  14 (13.9)  8 (7.9) |
| **Number with perfect health** | 10 (9.9) |
|  |  |
| **Visual analogue health score**  Mean (95% CI)  Range | 60.6 (56.4-64.7)  5-100 |
| **EQ5D Value Index (0=as good as dead, 1 = perfect health)**  Mean (95% CI)  Range | 0.594 (95% CI 0.535-0.652)  -0.285-1.000 |

# Supplementary Table 3: Number completing physiological tests

6MWT: 6-minute walking test; BD: bronchodilator; FeNO: exhaled nitric oxide measurement

| **Condition** | **Number completed** | **Number not completed** | **Reasons for not completing (number)** |
| --- | --- | --- | --- |
| **6MWT** | 72 | 29 | Too breathless: 12  Unable to complete 6 minutes’ walking: 12  Oxygen levels too low: 3  Unwilling: 2 |
| **Any Spirometry** | 101 | 0 |  |
| Pre- & post-BD spirometry | 65 | 36 | Pre-BD spirometry only: 19  Post-BD spirometry only: 17 |
| Pre-BD spirometry only | 19 |  | Poor cooperation: 9  Too breathless: 7  Cough: 1  Chest pain: 1  Unknown: 1 |
| Post-BD spirometry only | 17 |  | Self-administered BD before test: 17 |
| **Any full lung function** | 83 | 18 | Poor cooperation: 7  Too breathless: 8  Cough: 2  Chest pain: 1 |
| Only pre-BD full lung function | 7 |  | Poor cooperation: 3  Too breathless: 3  Unknown: 1 |
| Only post-BD full lung function | 15 |  | Self-administered BD before test: 14  Unknown: 1 |
| **FeNO** | 65 | 36 | Participant unable to use (10 attempts): 33  Too breathless: 2  Technical malfunction: 1 |

# Supplementary Table 4: DLCO results

Post-bronchodilator DLCO measurements, n=44.

DLCO: transfer factor, corrected for haemoglobin; LLN: lower limit of normal; SD: standard deviation; 95% CI: 95% confidence interval

|  | **DLCO ml/min/mmHg** | **% predicted** | **Z-score** | **Lower DLCO** |
| --- | --- | --- | --- | --- |
| **Mean:**  **SD:**  **95% CI (mean):**  **Range:** | 14.2  6.3  12.3-16.1  4.8-32.3 | 63.1  27.2  54.8-71.4  22.6-115.9 | -3.07  2.43  -3.81- -2.33  -7.81-1.11 | DLCO < LLN 29 (65.9%)  DLCO < 75% predicted  29 (65.9%)  DLCO < 40% predicted  11 (25.0%) |

# Supplementary Table 5: FeNO results

N=65

FeNO: exhaled nitric oxide; ppb: parts per billion; IQR: inter-quartile range.

|  | **FeNO ppb** | **Number of attempts** |
| --- | --- | --- |
| **Median:**  **IQR:**  **Range:** | 20  13-41  0-268 | 4  2-5  1-10 |

# Supplementary Table 6: Laboratory results

N=101. Participants could be reactive to multiple aeroallergens.

SD: standard deviation; 95% CI: 95% confidence interval.

|  | N (%) unless otherwise indicated |
| --- | --- |
| **White cell count (10^9/L)**  Mean  SD  Range | **10^9/L**  8.77  3.38  3.46-22.54  Missing: 1 |
| **Absolute eosinophil count (10^9/L)**  Mean  SD  95% CI  Range | **10^9/L**  0.22  0.25  0.17-0.27  0-1.40  Missing: 1 |
| Absolute eosinophils ≥300 cells/microlitre | 19 (19.0) |
| **Total IgE (IU/ml)**  Mean  SD  95% CI  Range | **(IU/ml)**  410.2  875.4  237.4-583.0  1-7193  Missing: 0 |
| Total IgE > 100IU/ml | 50 (49.5) |
| **Aeroallergen screen** |  |
| Aeroallergen screen positive | 47 (46.5) |
| *D. pteronyssinus* reactive | 35 (74.5) |
| *Blomia tropicalis* reactive | 32 (68.1) |
| Bermuda grass reactive | 19 (40.4) |
| Rye grass reactive | 25 (53.2) |
| *Cladosporium herbarum* reactive | 7 (14.9) |
| *Aspergillus fumigates* reactive | 7 (14.9) |
| *Alternaria alternata* reactive | 5 (10.6) |
| Cat dander reactive | 7 (14.9) |
| Dog dander reactive | 11 (23.4) |
| **Sputum** |  |
| Sputum given | 68 (67.3) |
| Sputum eosinophils present | 32/68 (46.4) |
| Description of sputum eosinophils | Scanty: 16/32 (50.0)  Moderate: 9/32 (28.1)  Numerous: 7/32 (21.9) |

# Supplementary Table 7: Correlations

FEV1 reversibility: Improvement in forced expiratory volume in 1 second by >200ml and ≥12%; PEF reversibility: improvement in peak expiratory flow by >20%; p: p-value; OR: odds ratio; 95% CI: 95% CI: 95% confidence interval; FeNO: exhaled nitric oxide; 6MWD: 6-minute walking distance; IgE: immunoglobulin E.

| **Variables** | **Test applied** | **Test result** |
| --- | --- | --- |
| Serum eosinophil count (10^9/L) and positive aeroallergen screen | Mann-Whitney U | W=901.5  p=0.01* |
| Serum eosinophil count (10^9/L) and total IgE (IU/ml) | Spearman’s correlation | Coefficient: 0.14 |
| Serum eosinophil count (10^9/L) and FeNO (ppb) | Spearman’s correlation | Coefficient: 0.24 |
| Serum eosinophil count (10^9/L) and sputum eosinophilia | Mann-Whitney U | W=631  P=0.64 |
| FEV1 reversibility and PEF reversibility | Fisher’s exact test | p=0.002*  OR (95% CI): 15.58 (2.73-88.86) |
| FEV1 (L/min) and 6MWD (metres) | Spearman’s correlation | Coefficient: 0.34 |
| FEV1 (L/min) and FeNO (ppb) | Spearman’s correlation | Coefficient: 0.30 |
| FEV1 (L/min) and serum eosinophil count (10^9/L) | Spearman’s correlation | Coefficient: 0.23 |
| FEV1 (L/min) and total IgE (IU/ml) | Spearman’s correlation | Coefficient: 0.24 |
| FEV1 (L/min) and positive aeroallergen screen | Mann-Whitney U | W=634  p=0.06 |
| Total IgE (IU/ml) and FeNO (ppb) | Spearman’s correlation | Coefficient: 0.10 |

# Supplementary Table 8: Univariate analysis for serum eosinophil count >300 cells/microlitre

N=101. 82 had total serum eosinophil count <300 cells/microlitre; 19 had total serum eosinophil count ≥300 cells/microlitre. Number (%) unless otherwise indicated.

Eos: total serum eosinophil count; IQR: inter-quartile range; p: p-value; OR: odds ratio; 95% CI: 95% CI: 95% confidence interval; mMRC: modified Medical Research Council dyspnoea score; FeNO: exhaled nitric oxide; ppb: parts per billion; 6MWD: 6-minute waking distance; FEV1: Forced expiratory volume in 1 second; FVC: forced vital capacity; PEF: peak expiratory flow; IgE: immunoglobulin E.

| **Variable** | **Descripton** | **Eos <300 cells/µL** | **Eos >= 300 cells/µL** | **Test applied**  **Result**  **p-value** |
| --- | --- | --- | --- | --- |
| **Age (years)** | Median  IQR | 60  51-65 | 53  35-62 | Mann-Whitney U  W=990.5  p=0.07 |
| **Sex** | Male | 36 (35.6) | 14 (13.9) | Chi-Square 5.47  OR (95% CI): 0.27 (0.09-0.85)  p=0.02* |
|  | Female | 46 (45.5) | 5 (5.0) |  |
| **Smoking status** | Current | 39 (38.6) | 9 (8.9) | Fisher’s exact test  p=0.88 |
|  | Ex | 33 (32.7) | 7 (6.9) | OR (95% CI) Current-Ex: 0.92 (0.31-2.74) |
|  | Never | 10 (9.9) | 3 (3.0) | OR (95% CI) Current-Never: 1.30 (0.30-5.71) |
| **Number of pack years smoked** | Median IQR | 23.3  (8.3-32.8) | 21.5  (9.4-25.7) | Mann-Whitney U  W=565.5  p=0.91 |
| **Number of exacerbations in last year** | Median  IQR | 3.5  2.0-6.0 | 3.0  1.5-4.0 | Mann-Whitney U  W=905.5  p=0.27 |
| **HIV status** | Negative | 64 (63.4) | 16 (15.8) | Fisher’s exact test  p=0.76 |
|  | Positive | 18 (17.8) | 3 (3.0) | OR (95% CI): 0.67 (0.17-2.54) |
| **History of tuberculosis** | No | 41 (41.0) | 13 (13.0) | Chi-square: 1.31  p=0.25  Missing: 1 |
|  | Yes | 40 (40.0) | 6 (6.0) | OR (95% CI): 0.47 (0.16-1.37) |
| **Any allergic co-morbidities** | No | 59 (58.4) | 11 (10.9) | Chi-square: 1.43  p=0.23 |
|  | Yes | 23 (22.8) | 8 (7.9) | OR (95 %CI): 1.87 (0.67-5.22) |
| **Family history of allergy** | No | 46 (45.5) | 9 (8.9) | Chi-square: 0.47  p=0.49 |
|  | Yes | 36 (35.6) | 10 (9.9) | OR (95 %CI): 1.42 (0.52-3.86) |
| **mMRC score** | 0 | 6 (5.9) | 3 (3.0) | Fisher’s exact test  P=0.33 |
|  | 1 | 2 (2.0) | 1 (1.0) | OR (95 %CI) 0-1: 1.00 (0.06-15.99) |
|  | 2 | 6 (5.9) | 2 (2.0) | OR (95 %CI) 0-2: 0.67 (0.08-5.54) |
|  | 3 | 13 (12.9) | 4 (4.0) | OR (95 %CI) 0-3: 0.61 (0.10-3.66) |
|  | 4 | 55 (54.5) | 9 (8.9) | OR (95 %CI) 0-4: 0.33 (0.07-1.55) |
| **FeNO (ppb)** | Median  IQR | 34  13-36 | 40  13-152 | Mann-Whitney U test  W=313.5  p=0.29  Missing: 35 |
| **FeNO >50ppb** | No | 41 (62.1) | 10 (15.2) | Fisher’s exact test  p=0.30  Missing: 35 |
|  | Yes | 10 (15.2) | 5 (7.6) | OR 2.05 (95 %CI): (0.57-7.35) |
| **6MWD (metres)** | Median  IQR | 365  293-439 | 410  355-455 | Mann-Whitney U test  W=359  p=0.23  Missing: 29 |
| **FEV1 (L/min)** | Median  IQR | 0.95  0.68-1.67 | 1.25  1.13-1.97 | Mann-Whitney U test  W=470  p=0.16  Missing: 19 |
| **FVC (L/min)** | Median  IQR | 2.28  1.85-2.87 | 2.81  2.37-3.24 | Mann-Whitney U test  W=430.5  p=0.07  Missing: 19 |
| **FEV1/FVC** | Median  IQR | 0.49  0.33-0.70 | 0.49  0.40-0.63 | Mann-Whitney U test  W=569  p=0.75  Missing: 19 |
| **FEV1/FVC < LLN** | No | 16 (19.5) | 3 (3.7) | Fisher’s exact test  P=0.54  Missing: 19 |
|  | Yes | 47 (57.3) | 16 (19.5) | OR (95% CI): 1.82 (0.47-7.06) |
| **FEV1/FVC < 0.7** | No | 16 (19.5) | 3 (3.7) | Fisher’s exact test  P=0.54  Missing: 19 |
|  | Yes | 47 (57.3) | 16 (19.5) | OR (95% CI): 1.82 (0.47-7.06) |
| **FEV1 reversibility (>12% + >200ml)** | No | 46 (70.8) | 12 (18.5) | Fisher’s exact test  P=1.0  Missing: 36 |
|  | Yes | 6 (9.2) | 1 (1.5) | OR (95% CI): 0.64 (0.07-5.83) |
| **PEF reversibility (>20%)** | No | 36 (55.4) | 11 (16.9) | Fisher’s exact test  P=0.32  Missing: 36 |
|  | Yes | 16 (24.6) | 2 (3.1) | OR (95% CI): 0.41 (0.08-2.06) |
| **Total IgE (IU/ml)** | Median  IQR | 80.0  29.6-314.3 | 266.0  131.0-864.5 | W=466  p=0.007* |
| **Total IgE >100 IU/ml** | No | 47 (46.5) | 4 (4.0) | Chi-square: 8.12  p=0.005* |
|  | Yes | 35 (34.7) | 15 (14.9) | OR (95% CI): 5.04 (1.54-16.50) |
| **Aeroallergen screen positive** | No | 49 (48.5) | 5 (5.0) | Chi-square: 6.93  p=0.008* |
|  | Yes | 33 (32.7) | 14 (13.9) | OR (95% CI): 4.16 (1.37-12.65) |
| **Sputum eosinophils present** | No | 30 (43.5) | 7 (10.1) | Chi-square  p=0.72  Missing: 32 |
|  | Yes | 27 (39.1) | 5 (7.2) | OR (95% CI): 0.79 (0.22-2.80) |

# Supplementary Table 9: Univariate analysis for FEV1 improvement after bronchodilator administration

N=101. 7 had an improvement of >200ml and ≥12% in FEV1 (forced expiratory volume in 1 second) after bronchodilator administration, “reversibility”. Total number unless otherwise indicated.

IQR: inter-quartile range; p: p-value; OR: odds ratio; 95% CI: 95% CI: 95% confidence interval; mMRC: modified Medical Research Council dyspnoea score; FeNO: exhaled nitric oxide; ppb: parts per billion; 6MWD: 6-minute waking distance; FEV1: Forced expiratory volume in 1 second; FVC: forced vital capacity; PEF: peak expiratory flow; IgE: immunoglobulin E.

| **Variable** | **Description** | **No FEV1 reversibility** | **FEV1 reversibility** | **Test applied**  **Result**  **p-value** |
| --- | --- | --- | --- | --- |
| **Age (years)** | Median  IQR | 60  50-65 | 52  43-59 | Mann-Whitney U test  W=425.5  p=0.20 |
| **Sex** | Male | 49 | 1 | Fisher’s exact test  P=0.11 |
|  | Female | 45 | 6 | OR (95% CI): 6.53 (0.86-56.39) |
| **Smoking status** | Current | 44 | 4 | Fisher’s exact test  p=0.19 |
|  | Ex | 39 | 1 | OR (95% CI) Current-ex: 0.28 (0.03-2.63) |
|  | Never | 11 | 2 | OR (95% CI) Current-Never: 2.00 (0.32-12.36) |
| **Number of pack years smoked** | Median IQR | 20  7-34 | 16  10-19 | Mann-Whitney U test  W=243  p=0.53 |
| **Number exacerbations in last year** | Median  IQR | 3  2-6 | 4  2-9 | Mann-Whitney U test  W=295  p=0.65 |
| **HIV status** | Negative | 74 | 6 | Fisher’s exact test  p=1.00 |
|  | Positive | 20 | 1 | OR (95% CI): 0.62 (0.07-5.42) |
| **History of tuberculosis** | No | 50 | 4 | Fisher’s exact test  p=1.00  Missing: 1 |
|  | Yes | 43 | 3 | OR (95% CI): 0.87 (0.18-4.11) |
| **Any allergic co-morbidities** | No | 65 | 5 | Fisher’s exact test  p=1.00 |
|  | Yes | 29 | 2 | OR (95% CI): 0.90 (0.16-4.89) |
| **Family history of allergy** | No | 52 | 3 | Fisher’s exact test  p=0.70 |
|  | Yes | 42 | 4 | OR (95% CI): 1.65 (0.35-7.79) |
|  |  |  |  |  |
| **mMRC score** | 0 | 7 | 2 | Fisher’s exact test  P=0.20 |
|  | 1 | 3 | 0 |  |
|  | 2 | 7 | 1 |  |
|  | 3 | 17 | 0 |  |
|  | 4 | 60 | 4 |  |
| **FeNO (ppb)** | Median  IQR | 19  13-41 | 62  42-114 | Mann-Whitney U test  W=41  p=0.10  Missing: 35 |
| **FeNO >50ppb** | No | 50 | 1 | Fisher’s exact test  p=0.13  Missing: 35 |
|  | Yes | 13 | 2 | OR (95% CI): 7.69 (0.65-91.6) |
| **6MWD (metres)** | Median  IQR | 384  300-440 | 420  363-439 | Mann-Whitney U test  W=154  p=0.77 |
| **Serum eosinophil count (10^9/L)** | Median  IQR | 145  70-258 | 130  80-240 | Mann-Whitney U test  W=318.5  p=0.89 |
| **Serum eosinophils ≥300 cells/microlitre** | No | 76 | 6 | Fisher’s exact test  p=1.0 |
|  | Yes | 18 | 1 | OR (95% CI): 0.70 (0.08-6.22) |
| **Total IgE (IU/ml)** | Median  IQR | 90.5  31.2-351.8 | 317.0  110.8-564.5 | Mann-Whitney U test  W=240.5  p=0.24 |
| **Total IgE >100 IU/ml** | No | 49 | 2 | Fisher’s exact test  p=0.27 |
|  | Yes | 45 | 5 | OR (95% CI): 2.72 (0.50-14.74) |
| **Aeroallergen screen positive** | No | 52 | 2 | Fisher’s exact test  p=0.25 |
|  | Yes | 42 | 5 | OR (95% CI): 3.10 (0.57-16.77) |
| **Sputum eosinophils present** | No | 32 | 5 | Fisher’s exact test  p=0.06  Missing: 32 |
|  | Yes | 32 | 0 |  |

# Supplementary Table 10: Univariate analysis for PEF improvement after bronchodilator administration

N=101. 18 had an improvement of >20% in PEF (peak expiratory flow) after bronchodilator administration. Total number unless otherwise indicated.

Eos: total serum eosinophil count; IQR: inter-quartile range; p: p-value; OR: odds ratio; 95% CI: 95% CI: 95% confidence interval; mMRC: modified Medical Research Council dyspnoea score; FeNO: exhaled nitric oxide; ppb: parts per billion; 6MWD: 6-minute waking distance; FEV1: Forced expiratory volume in 1 second; FVC: forced vital capacity; PEF: peak expiratory flow; IgE: immunoglobulin E.

| **Variable** |  | **No >20% PEF improvement** | **PEF improvement >20%** | **Test applied**  **Result**  **p-value** |
| --- | --- | --- | --- | --- |
| **Age (years)** | Median  IQR | 59  49-65 | 61  54-66 | Mann-Whitney U test  W=647  p=0.38 |
| **Sex** | Male | 45 | 5 | Chi square  p=0.04* |
|  | Female | 38 | 13 | OR (95% CI): 3.08 (1.01-9.42) |
| **Smoking status** | Current | 41 | 7 | Fisher’s exact test  p=0.67 |
|  | Ex | 32 | 8 | OR (95% CI): current-ex 1.46 (0.48-4.64) |
|  | Never | 10 | 3 | OR (95% CI): current-never 1.76 (0.38-8.03) |
| **Number of pack years smoked** | Median IQR | 23  9-35 | 20  11-24 | Mann-Whitney U test  W=547  p=1.0 |
| **Number of exacerbations in last year** | Median  IQR | 3  2-4 | 3  2-5 | Mann-Whitney U test  W=778.5  p=0.78 |
| **HIV status** | Positive | 17 | 4 | Fisher’s exact test  p=1.00 |
|  | Negative | 66 | 14 | OR (95% CI): 1.11 (0.32-3.80) |
| **History of tuberculosis** | No | 43 | 11 | Chi square  p=0.45  Missing: 1 |
|  | Yes | 39 | 7 | OR (95% CI): 0.7 (0.25-1.99) |
| **Any allergic co-morbidities** | No | 57 | 13 | Chi square  0.77 |
|  | Yes | 26 | 5 | OR (95% CI): 0.84 (0.27-2.61) |
| **Family history of allergy** | No | 49 | 6 | Chi square  p=0.047* |
|  | Yes | 34 | 12 | OR (95% CI): 2.88 (0.99-8.43) |
| **mMRC** | 0 | 9 | 0 | Fisher’s exact test  P=0.41 |
|  | 1 | 2 | 1 |  |
|  | 2 | 6 | 2 |  |
|  | 3 | 15 | 2 |  |
|  | 4 | 51 | 13 |  |
| **FeNO (ppb)** | Median  IQR | 59  49-65 | 61  54-66 | Mann-Whitney U test  W=434.5  p=0.07  Missing: 35 |
| **FeNO >50ppb** | No | 40 | 11 | Fisher’s exact test  p=0.27  Missing: 35 |
|  | Yes | 14 | 1 | OR (95% CI): 0.26 (0.03-2.20) |
| **6MWD (metres)** | Median  IQR | 392  302-448 | 364  310-420 | Mann-Whitney U test  W=439  p=0.64 |
| **Serum eosinophil count (10^9/L)** | Median  IQR | 150  75-270 | 125  43-198 | W=861  p=0.31 |
| **Serum eosinophils ≥300 cells/microlitre** | No | 66 | 16 | Fisher’s exact test  p=0.51 |
|  | Yes | 17 | 2 | OR (95% CI): 0.49 (0.10-2.32) |
| **Total IgE (IU/ml)** | Median  IQR | 90.9  35.8-380.0 | 122  31.5-429.0 | Mann-Whitney U test  W=731.5  p=0.89 |
| **Total IgE >100 IU/ml** | No | 43 | 8 | Chi square  p=0.57 |
|  | Yes | 40 | 10 | OR (95% CI): 1.34 (0.48-3.74) |
| **Aeroallergen screen positive** | No | 45 | 9 | Chi square  p=0.75 |
|  | Yes | 38 | 9 | OR (95% CI): 1.18 (0.43-3.28) |
| **Sputum eosinophils present** | No | 30 | 7 | Fisher’s exact test  p=0.16  Missing: 32 |
|  | Yes | 30 | 2 | OR (95% CI): 0.29 (0.05-1.49) |

# Supplementary Table 11: Univariate analysis for death

N=220. 40 died in the study period. Total number unless otherwise indicated.

IQR: inter-quartile range; p: p-value; OR: odds ratio; 95% CI: 95% CI: 95% confidence interval; SABA: short-acting beta-agonist inhaler; ICS: inhaled corticosteroid; HIV: human immunodeficiency virus infection.

| **Variable** |  | **Survived** | **Died** | **Test applied**  **Result**  **p-value** |
| --- | --- | --- | --- | --- |
| **Age (years)** | Median  IQR | 59  48-64 | 62  50-67 | Mann-Whitney U test  W=3026  p=0.11 |
| **Sex** | Male | 89 | 24 | Chi square 1.46  p=0.23 |
|  | Female | 91 | 16 | OR (95% CI): 0.65 (0.32-1.31) |
| **Employment** | Employed | 29 | 2 | Fisher’s exact test  p=0.11 |
|  | Pensioner | 74 | 22 |  |
|  | Unemployed | 77 | 16 |  |
| **Smoking status** | Current | 82 | 20 | Fisher’s exact test  p=0.74 |
|  | Ex | 71 | 16 |  |
|  | Never | 27 | 4 |  |
| **Number of pack years smoked** | Median  IQR | 18.5  8-35 | 27  15-44 | Mann-Whitney U test  W=2054  p=0.03* |
| **Asthma diagnosis** | Yes | 74 | 7 | Chi-squared  p=0.005* |
|  | No | 106 | 33 | OR (95% CI): 3.29 (1.38-7.84) |
| **PTLD diagnosis** | Yes | 13 | 7 | Chi-squared  p=0.04* |
|  | No | 167 | 33 | OR (95% CI): 0.37 (0.14-0.99) |
| **COPD diagnosis** | Yes | 100 | 29 | Chi-squared  p=0.049* |
|  | No | 80 | 11 | OR (95% CI): 0.47 (0.22-1.01) |
| **Bronchiectasis diagnosis** | Yes | 7 | 4 | Fisher’s exact test  p=0.12 |
|  | No | 173 | 36 | OR (95% CI): 0.36 (0.10-1.31) |
| **SABA use** | No | 35 | 14 | Chi-square  p=0.03* |
|  | Yes | 145 | 26 | OR (95% CI): 0.45 (0.21-0.95) |
| **Any ICS use** | No | 134 | 33 | Chi-square  p=0.28 |
|  | Yes | 46 | 7 | OR (95% CI): 0.62 (0.26-1.49) |
| **Exacerbation history** | Yes | 25 | 5 | Fisher’s exact test  P=1.00 |
|  | No | 155 | 35 | OR (95% CI): 1.13 (0.40-3.16) |
| **Number of exacerbations in last year** | Median  IQR | 3  1-5 | 3  2-6 | Mann-Whitney U test  W=3334.5  p=0.46 |
| **In hospital for exacerbations** | No | 27 | 12 | Chi-square  p=0.03* |
|  | Yes | 136 | 25 | OR (95% CI): 0.41 (0.19-0.92) |
| **>2 exacerbations in last year** | No | 92 | 18 | Chi-square  p=0.48 |
|  | Yes | 88 | 22 | OR (95% CI): 1.28 (0.64-2.54) |
| **HIV status** | Positive | 33 | 7 | Fisher’s exact test  p=0.97  Missing: 5 |
|  | Negative | 144 | 31 | OR (95% CI): 1.01 (0.41-2.50) |
| **History of tuberculosis** | No | 102 | 17 | Chi square  p=0.17  Missing: 3 |
|  | Yes | 77 | 21 | OR (95% CI): 1.64 (0.81-3.31) |
| **Diabetes** | Yes | 36 | 6 | Chi-square  p=0.47 |
|  | No | 144 | 34 | OR (95% CI): 1.42 (0.55-3.63) |
| **Hypertension** | Yes | 105 | 27 | Chi-square  P=0.28 |
|  | No | 75 | 13 | OR (95% CI): 0.67 (0.32-1.39) |
| **Cardiac disease** | Yes | 35 | 14 | Chi-square  p=0.03* |
|  | No | 145 | 26 | OR (95% CI): 0.44 (0.21-0.95) |
| **Number of co-morbidities** | Median  IQR | 1  1-2 | 2  1-3 | Mann-Whitney U test  W=3061  p=0.13 |
| **Any allergic co-morbidities** | No | 128 | 34 | Chi square  p=0.07 |
|  | Yes | 52 | 6 | OR (95% CI): 0.43 (0.17-1.10) |

# Supplementary Table 12: Univariate analysis for re-admissions

N=220. 89 were re-admitted during the study period. Total number unless otherwise indicated.

IQR: inter-quartile range; p: p-value; OR: odds ratio; 95% CI: 95% CI: 95% confidence interval; SABA: short-acting beta-agonist inhaler; ICS: inhaled corticosteroid; HIV: human immunodeficiency virus infection.

| **Variable** |  | **Not re-admitted** | **Re-admitted** | **Test applied**  **Result**  **p-value** |
| --- | --- | --- | --- | --- |
| **Age (years)** | Median  IQR | 60  50-65 | 57  47-65 | Mann-Whitney U test  W=5985  p=0.74 |
| **Sex** | Male | 66 | 47 | Chi square 1.46  p=0.72 |
|  | Female | 65 | 42 | OR (95% CI): 0.91 (0.53-1.56) |
| **Employment** | Employed | 27 | 4 | Fisher’s exact test  p=0.0001* |
|  | Pensioner | 61 | 35 | OR (95% CI) employed-pensioner: 3.87 (1.25-11.98) |
|  | Unemployed | 43 | 50 | OR (95% CI) pensioner-unemployed: 2.03 (1.13-3.63) |
| **Smoking status** | Current | 57 | 45 | Fisher’s exact test  p=0.33 |
|  | Ex | 52 | 35 |  |
|  | Never | 22 | 9 |  |
| **Number of pack years smoked** | Median  IQR | 19  9-35 | 23  9-38 | Mann-Whitney U test  W=4023  p=0.42 |
| **Asthma diagnosis** | Yes | 52 | 29 | Chi-squared  p=0.28 |
|  | No | 79 | 60 | OR (95% CI): 1.36 (0.77-2.40) |
| **PTLD diagnosis** | Yes | 11 | 9 | Chi-squared  p=0.66 |
|  | No | 120 | 80 | OR (95% CI): 0.81 (0.32-2.06) |
| **COPD diagnosis** | Yes | 71 | 58 | Chi-squared  p=0.10 |
|  | No | 60 | 31 | OR (95% CI): 0.63 (0.36-1.01) |
| **Bronchiectasis diagnosis** | Yes | 3 | 8 | Fisher’s exact test  p=0.05 |
|  | No | 128 | 81 | OR (95% CI): 0.24 (0.06-0.92) |
| **SABA use** | No | 36 | 13 | Chi-square  p=0.02* |
|  | Yes | 95 | 76 | OR (95% CI): 2.22 (1.10-4.47) |
| **Any ICS use** | No | 100 | 67 | Chi-square  p=0.86 |
|  | Yes | 31 | 22 | OR (95% CI): 1.06 (0.57-1.98) |
| **Exacerbation history** | Yes | 24 | 6 | Fisher’s exact test  P=0.01* |
|  | No | 107 | 83 | OR (95% CI): 3.10(1.21-7.94) |
| **Number of exacerbations in last year** | Median  IQR | 3  1-5 | 4  2-6 | Mann-Whitney U test  W=4795.5  p=0.02* |
| **In hospital for exacerbations** | No | 25 | 14 | Chi-square  p=0.29 |
|  | Yes | 88 | 73 | OR (95% CI): 1.48 (0.72-3.06) |
| **>2 exacerbations in last year** | No | 64 | 46 | Chi-square  p=0.68 |
|  | Yes | 67 | 43 | OR (95% CI): 0.89 (0.52-1.53) |
| **HIV status** | Positive | 21 | 19 | Fisher’s exact test  p=0.32  Missing: 5 |
|  | Negative | 107 | 68 | OR (95% CI): 0.70 (0.35-1.40) |
| **History of tuberculosis** | No | 86 | 33 | Chi square  p=0.001*  Missing: 3 |
|  | Yes | 43 | 55 | OR (95% CI): 3.33 (1.89-5.87) |
| **Diabetes** | Yes | 24 | 18 | Chi-square  p=0.72 |
|  | No | 107 | 71 | OR (95% CI): 0.88 (0.44-1.75) |
| **Hypertension** | Yes | 81 | 51 | Chi-square  P=0.50 |
|  | No | 50 | 38 | OR (95% CI): 1.21 (0.70-2.09) |
| **Cardiac disease** | Yes | 31 | 18 | Chi-square  p=0.55 |
|  | No | 100 | 71 | OR (95% CI): 1.22 (0.63-2.36) |
| **Number of co-morbidities** | Median  IQR | 1  1-2 | 1  1-2 | Mann-Whitney U test  W=5822.5  p=0.99 |
| **Any allergic co-morbidities** | No | 92 | 70 | Chi square  p=0.16 |
|  | Yes | 39 | 19 | OR (95% CI): 0.64 (0.34-1.20) |

# Supplementary Figure 1: Number of diagnoses pre- and post-investigations

N=101.

PTLD: post-tuberculosis lung disease; COPD: chronic obstructive pulmonary disease.
